# Polymorphisms of the apolipoprotein E gene affect response to atorvastatin therapy in acute ischemic stroke

**DOI:** 10.3389/fcvm.2022.1024014

**Published:** 2022-11-08

**Authors:** QianQian Bi, XiaoYu Zhou, YanQin Lu, Wang Fu, YongPeng Wang, Feng Wang, Jue Wang

**Affiliations:** ^1^Department of Neurology, Seventh People’s Hospital of Shanghai University of Traditional Chinese Medicine, Shanghai, China; ^2^Department of Neurology, Shanghai Tenth People’s Hospital, Tongji University School of Medicine, Shanghai, China; ^3^Department of Infectious Diseases, Shanghai Tenth People’s Hospital, Tongji University School of Medicine, Shanghai, China

**Keywords:** apolipoprotein E, polymorphism, lipid-lowering, atherosclerosis, carotid artery plaques

## Abstract

**Background:**

Polymorphisms of the apolipoprotein E (APOE) gene are related to the efficacy of statin therapy. The biological functions of the APOE subtypes determine the metabolism of blood plasma lipids and the progression of atherosclerosis. This study aimed to explore the impact of APOE gene polymorphisms on the effect of atorvastatin on lipid regulation and plaque stabilization.

**Methods:**

The study was a prospective cohort study that consecutively included patients with acute ischemic stroke (AIS) in the Department of Neurology, Shanghai Tenth People’s Hospital, from December 2018 to December 2019. The patients were divided into E2, E3, and E4 groups according to their APOE genotype. Atorvastatin (20 mg) was administrated to all patients. Changes in blood lipid levels over 3 months and plaque size and stability over 12 months were analyzed.

**Results:**

We enrolled 253 consecutive patients with AIS, of whom, 136 had carotid atherosclerotic plaques. Two patients with genotype E2/E4 were excluded. There were 30 patients in the E2 group (12.0%), 191 patients in the E3 group (76.0%), and 30 patients in the E4 group (12.0%). The lowest percentage reduction in low-density lipoprotein cholesterol (LDL-C) was observed in the E4 group (41.2%), while the highest percentage reduction was observed in the E2 group (17.6%). The plaques in the E2 group showed slower progression, while those in the E4 group showed more rapid progression.

**Conclusion:**

APOE gene polymorphisms affect the biological functions of atorvastatin. Compared to the ε3 or ε4 allele, the ε2 allele exerted a greater lipid-lowering effect on LDL-C levels, enhanced the ability of atorvastatin to stabilize carotid artery plaques, and slowed carotid artery plaque progression.

## Introduction

Atherosclerosis is one of the leading causes of stroke. Low-density lipoprotein cholesterol (LDL-C) is closely related to the progression of atherosclerosis (1). With every 1 mmol/L reduction in LDL-C, the relative risk of stroke decreases by 21.1%, thus showing the importance of reducing LDL-C levels for stroke prevention (2). Statins that competitively inhibit critical enzymes in cholesterol synthesis are the most widely used lipid-lowering drugs and have become one of the three cornerstones of acute ischemic stroke (AIS) treatment. However, apparent individual differences in lipid-lowering effects have been observed with the widespread use of statins. Statin metabolism is affected by a variety of genes, and gene polymorphisms are related to the lipid-lowering effects of statins. Genetic factors contribute to approximately 70% of the efficacy of statin treatment (3), and the apolipoprotein E (APOE) gene is closely related (4, 5).

APOE is mainly synthesized in the periphery of the liver. On the one hand, as a structural protein of chylomicron (CM), LDL-C, very low-density lipoprotein cholesterol (VLDL-C), and part of high-density lipoprotein cholesterol (HDL-C), APOE is beneficial in stabilizing the structure of these lipoproteins. On the other hand, as a ligand and member of the LDL receptor family, it regulates blood CM, LDL-C, VLDL-C, and HDL-C levels (6, 7). Gene polymorphisms determine the transport and regulation of blood lipids. The gene coding APOE is located on chromosome 19 and is approximately 3.7 kb in length (8). It comprises two loci, rs429358T > C^3,937^ and rs7412C > T^4,075^, and includes four alleles, ε2 (T^3,937^–T^4,075^), ε3 (T^3,937^–C^4,075^), ε4 (C^3,937^–C^4,075^), and ε3r (C^3,937^–T^4,075^), of which ε3r is extremely rare. To date, only two Caucasian families in Italy and one Yoruba family in Ibadan have been reported with this allele, which is why it is generally excluded from clinical studies (9). The ε3 is the most common in the general population (10) and has a frequency of 85% in Asia, 82% in North America, and 77% in South America (11). ε3 is considered “wild type” due to its high frequency in the general population, while ε2 and ε4 alleles are mutations of ε3. The three alleles comprise six common genotypes, including three homozygotes (E2/E2, E3/E3, and E4/E4) and three heterozygotes (E2/E3, E2/E4, and E3/E4).

Due to differences in protein conformations, the affinities for cholesterol receptors in people with distinct genotypes are different, affecting the efficacy of statin therapy. Some studies have shown that statins may confer reduced benefits in APOE ε4 carriers (12) and that ε2 gene carriers may experience superior lipid-lowering effects (13). However, other studies have suggested that the APOE genotype is not significantly associated with the lipid-lowering effect of statins (14). In another study, polymorphisms of the APOE gene determined baseline LDL-C levels, but not the lipid-lowering effect of statins (15). In the Chilean population, patients with the E3/E4 genotype had a smaller reduction in cholesterol levels after statin therapy than those with the E3/E3 genotype (16). However, the association between APOE gene polymorphisms and the progression of atherosclerotic plaques with statin therapy is not well described. Thus, we aimed to explore the differences in lipid-lowering effects and the progression of atherosclerotic plaques with atorvastatin in different APOE genotypes.

## Materials and methods

### Patients and study design

We prospectively and consecutively collected data from a cohort that included all hospitalized patients with AIS in the Department of Neurology, Shanghai Tenth People’s Hospital, between December 2018 and December 2019. Patients were enrolled if they met the following criteria: (1) patients diagnosed with AIS using MRI within 7 days after stroke onset; (2) no previous history of lipid-lowering drug use, such as statins, fibrins, and PCSK9 inhibitors, or lipid-lowering drug withdrawal for more than 1 month; (3) willing to receive 20 mg atorvastatin; (4) patients gave informed consent and participated voluntarily. Exclusion criteria included (1) severe liver or kidney dysfunction or major cardiovascular or respiratory diseases; (2) allergic or intolerant to atorvastatin; (3) severe trauma or major surgery recently; (4) patients with non-compliance or poor compliance. The genotype was confirmed at the study’s inception. Previous studies have indicated that the E2 and E4 mutant alleles may have opposite effects on treatment. Therefore, we excluded patients with the E2/E4 genotype. Patients were divided into three groups according to their APOE genotype: E2 (E2/E2 + E2/E3), E3 (E3/E3), and E4 (E3/E4 + E4/E4). All eligible patients received atorvastatin (atorvastatin calcium tablets, Pfizer Pharmaceuticals Limited, 20 mg*7) 20 mg daily and were followed up for 3 months. Patients with carotid plaques were followed up for 12 months. Blood lipids were tested at 3 months and carotid artery ultrasonography was performed at 12 months. The basic characteristics of every patient, including gender, age, height, weight, past medical history, and personal history, were recorded from the electronic medical records and by direct communication with the patients by two doctors at the beginning of the study, and the information was double checked by two doctors at the end.

All patients provided written informed consent to participate in this study. The Ethics Committee of Shanghai Tenth People’s Hospital approved this study (No. 22k205).

### Apolipoprotein E genotyping

Genomic DNA was extracted from whole blood using the whole blood DNA Extraction Kit (Beijing Jingzhun Medical Technology Co., Ltd.). Polymerase chain reaction [Honglong Biotechnology (Shanghai) Co., Ltd.] was used for DNA amplification, and the product fragments were subjected to capillary electrophoresis sequencing analysis. According to the peak characteristics performing APOE genotyping.

### Detection and classification of plaques

Two ultrasound specialists performed carotid ultrasonography using a color Doppler ultrasound system (Logiq E9, GE, USA). Each patient was placed in a quiet supine position, with the head tilted back and turned to the opposite side. The probe was not pressurized, following the lateral border of the sternocleidomastoid muscle from bottom to top. The patient’s common carotid artery trunk, common carotid artery bifurcation, and neck were observed in turn. The internal and external carotid arteries were observed and recorded for plaque length, thickness, and echogenicity. Information on the largest plaque was recorded if a patient had multiple plaques simultaneously. Carotid artery intima-media thickness (CIMT) was measured in a 1 cm segment at the bulb of the common carotid artery and 1 cm each from its proximal and distal segments, and the average value of three points was taken as the final value of CIMT. The contralateral side was observed in the same way.

Atherosclerotic plaque formation is defined as a condition in which the intima-media thickness (IMT) is ≥ 1.5 mm, and it protrudes from the vascular lumen, or the localized intimal thickening is > 50% of the surrounding IMT. According to the morphology and echo characteristics of ultrasound, plaques can be divided into (I) hypoechoic lipid soft plaques, (II) fibrous flat plaques with medium echoes rich in collagen tissue, (III) hyperechoic calcifications with acoustic shadows, and (IV) ulcerative mixed plaques with varying echo intensity (17). Among them, (I), (II), and (IV) are vulnerable plaques, and (III) are stable plaques.

### Statistical analysis

Statistical analyses were performed using IBM SPS Statistics (version 26.0; IBM, Armonk, NY, USA). Normally distributed measurement data are presented as mean ± standard deviation, and non-normally distributed data are expressed as median and quartile. To compare the three groups, one-way analysis of variance or the Kruskal Wallis test was used, and differences between the two groups were analyzed using the independent samples *T*-test or the Mann-Whitney *U*-test. The count data are expressed as frequency (percentage), and differences between groups were compared using the chi-square test. To explore the factors affecting LDL-C reduction and changes in plaque length, univariate and multivariate linear regression analyses were performed. The Hardy-Weinberg genetic balance test of APOE was performed using the chi-square test. A two-tailed value of *p* < 0.05 was considered statistically significant.

## Results

### Polymorphisms of apolipoprotein E

**FIGURE 1:**
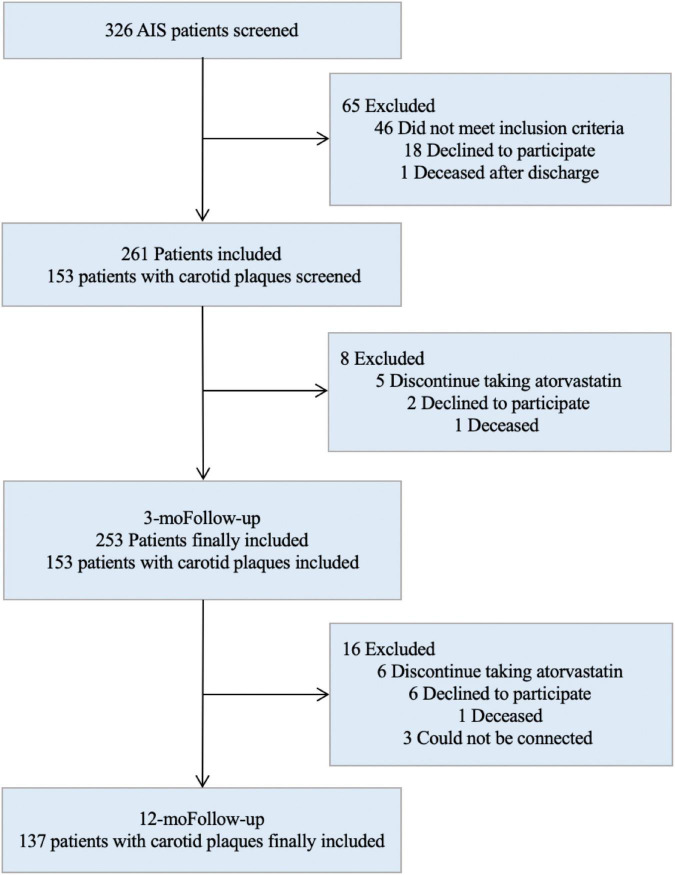
Screening flowchart.

A total of 253 AIS patients with complete follow-up data were included in the study (Figure 1). Six genotypes were detected: three cases with E2/E2 (1.2%), 27 cases with E2/E3 (10.7%), two cases with E2/E4 (0.8%), 191 cases with E3/E3 (75.5%), 28 cases with E3/E4 (11.1%), and two cases with E4/E4 (0.8%).

According to the Hardy-Weinberg genetic balance test, we calculated the theoretical frequency of the included population and compared it with the actual frequency in Table 1. The results indicated that the population in our study was in line with the Hardy-Weinberg genetic balance (*P* = 0.1486), which means that it had good group representation.

**TABLE 1:** The Hardy-Weinberg genetic balance test of the APOE gene.

Genotype	Actual frequency	Theoretical frequency	χ^2^	*P*-value
E2/E2	3 (1.2%)	1.21	3.8133	0.1486
E2/E3	27 (10.7%)	30.23		
E2/E4	2 (0.8%)	2.35		
E3/E3	191 (75.5%)	188.87		
E3/E4	28 (11.1%)	29.71		
E4/E4	2 (0.8%)	1.14		

Two patients with the E2/E4 genotype were excluded and the remaining 251 patients were divided into three groups: 30 patients in the E2 group (12%), 191 in the E3 group (76%), and 30 in the E4 group (12%). The baseline characteristics of the patients were similar among the three groups (Table 2).

**TABLE 2:** Characteristics of the patients at baseline.

Variable		APOE genotype group			*P*-value
	
	Total group *n* = 251	E2 cases *n* = 30	E3 cases *n* = 191	E4 cases *n* = 30	
Gender, no. (%)					0.078
Male	186 (74.1)	20 (66.7)	148 (77.5)	18 (60.0)	
Female	65 (25.9)	10 (33.3)	43 (22.5)	12 (40.0)	
Age, median (IQR), y	65 (60.72)	65.5 (60.71)	66 (60.71)	65 (61.77)	0.073
Age, no. (%)					0.815
>60	181 (72.1)	22 (73.3)	136 (71.2)	23 (76.7)	
≤60	70 (27.9)	8 (26.7)	55 (28.8)	7 (23.3)	
Hypertension, no. (%)					0.120
Yes	172 (68.5)	16 (53.3)	133 (69.6)	23 (76.7)	
No	79 (31.5)	14 (46.7)	58 (30.4)	7 (23.3)	
Diabetes mellitus, no. (%)					0.124
Yes	97 (38.6)	7 (23.3)	80 (41.9)	10 (33.3)	
No	153 (61.0)	23 (76.7)	111 (58.1)	20 (66.7)	
CHD, no. (%)					0.592
Yes	33 (13.1)	5 (16.7)	23 (12.0)	5 (16.7)	
No	218 (86.9)	25 (83.3)	168 (88.0)	25 (83.3)	
AF, no. (%)					0.319
Yes	17 (6.8)	3 (10.0)	12 (6.3)	4 (13.3)	
No	234 (93.8)	27 (90.0)	179 (93.7)	26 (86.7)	
Smoking*, no. (%)					0.119
Yes	91 (36.3)	13 (43.3)	72 (37.7)	6 (20.0)	
No	160 (63.7)	17 (56.7)	119 (62.3)	24 (80.0)	
Drinking*, no. (%)					0.738
Yes	34 (13.5)	5 (16.7)	26 (13.6)	3 (10.0)	
No	217 (86.5)	25 (83.3)	165 (86.4)	27 (90.0)	
BMI, median (IQR), kg/m^2^	24.5 (22.9, 26.7)	24.2 (22.9, 25.7)	24.5 (22.9, 26.7)	24.7 (22.3, 28.7)	0.397
BMI, no. (%)					0.960
>24	148 (59.0)	18 (60.0)	113 (59.2)	17 (56.7)	
≤24	103 (41.0)	12 (40.0)	78 (40.8)	13 (43.3)	

Smoking*, smoked at least 100 cigarettes or 100 g of tobacco in their lifetime; Drinking*, ≥ 1 time per month, ≥ 1 standard drink each time, one standard drink equals 10 g of pure alcohol. AF, atrial fibrillation; CHD, coronary heart disease; BMI, body mass index.

### Effects on blood lipids and plaques

**FIGURE 2:**
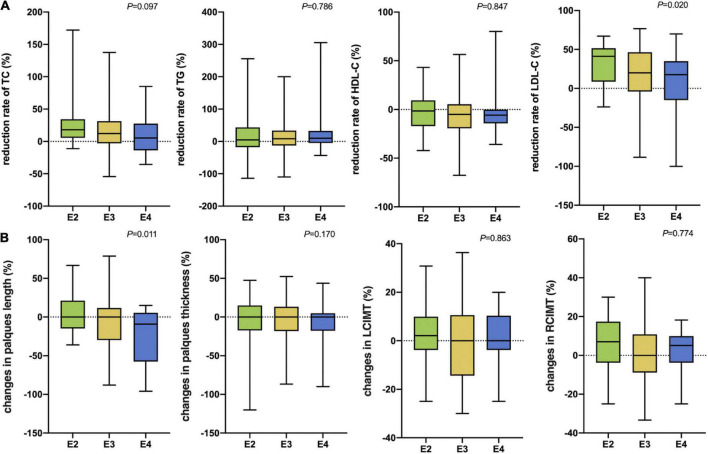
**(A)** The rate of reduction in blood lipids after 3 months of atorvastatin treatment in the three groups. **(B)** Changes in carotid plaque size after 12 months of atorvastatin treatment in the three groups. TC, Total cholesterol; TG, Triglyceride; HDL-C, High-density lipoprotein cholesterol; LDL-C, Low-density lipoprotein cholesterol; LCIMT, Left carotid intima-media thickness; RCIMT, Right carotid intima-media thickness.

After 3 months of atorvastatin treatment, total cholesterol (TC), triglyceride (TG), and LDL-C levels were lower than baseline. There was a significant difference in the reduction rate of blood LDL-C level after treatment (Figure 2A); the E2 group had the highest reduction rate (41.2%), followed by the E3 group (19.8%), while the E4 group had the lowest (17.6%) (*P* = 0.020). When we stratified the cohort by gender, no differences were found in females (Table 3). We performed multiple linear regression based on univariate analysis and expertise (Tables 4, 5). The results showed that the E2 group was sensitive to atorvastatin therapy based on the LDL-C reduction rate (Table 6). The progression of carotid plaques in the E4 group was more rapid than in the E2 and E3 groups (*P* = 0.011) (Figure 2B). After 12 months of atorvastatin treatment, the percentage of vulnerable plaques decreased in the three groups, with significant plaque stabilization in the E2 group (Table 7).

**TABLE 3:** Percentage variation in lipid concentrations after treatment with atorvastatin and stratified analyses according to gender.

	E2 *n* = 30, male = 20, female = 10	E3 *n* = 191, male = 148, female = 43	E4 *n* = 30, male = 18, female = 12	*P*-value
**TC**				
All	–19.3 (–36.6, –5.9)	–5.9 (–31.2, 3.1)	–4.9 (–25.2, 12.8)	0.097
Male	–19.5 (–38.2, –5.2)	–12.6 (–31.3, 2.8)	–13.9 (–27.4, 5.9)	0.312
Female	–19.4 (–21.0, –8.1)	–14.5 (–30.5, 14.7)	6.4 (–18.1, 25.4)	0.252
**TG**				
All	–6.2 (–35.3, 24.7)	24.7 (–25.4, 16.5)	–10.0 (–34.8, 4.6)	0.786
Male	0.0 (–31.3, 27.6)	–6.7 (–25.6, 16.5)	–10.0 (–29.6, 0.0)	0.681
Female	–12.3 (–40.3, 14.0)	–8.8 (–23.3, 19.3)	–7.5 (–39.5, 23.4)	0.921
**HDL-C**				
All	2.7 (–8.0, 16.8)	16.8 (–5.2, 18.5)	5.9 (0.0, 12.0)	0.847
Male	3.4 (–9.4, 23.6)	3.6 (–5.7, 17.9)	5.9 (0.0, 11.8)	0.983
Female	1.4 (–0.7, 14.4)	9.4 (0.0, 20.0)	3.7 (–19.7, 20.5)	0.493
6				
**LDL-C**				
All	–41.2 (–52.2, –10.4)	–19.7 (–46.7, 1.9)	–17.6 (–36.6, 14.8)	0.020
Male	–48.5 (–55.5, –9.8)	–19.7 (–46.7, 1.5)	–23.2 (–37.3, 1.2)	0.037
Female	–36.9 (–41.2, –21.5)	–20.8 (–43.8, 16.1)	0.0 (–34.0, 71.0)	0.316

Median of the individual percentage changes (IQR).

**TABLE 4:** Analysis of factors influencing the LDL-C reduction rate following atorvastatin therapy.

	B	B (95%CI) lower limit	B (95%CI) upper limit	*P*
Gender	–9.35	–22.31	3.61	0.157
Age	0.07	–0.46	0.60	0.794
Hypertension	4.34	–7.92	16.61	0.486
Diabetes mellitus	8.16	–3.41	19.72	0.166
CHD	12.80	–3.91	29.52	0.133
AF	38.04	16.89	59.19	<0.001
Smoking	1.87	–9.84	13.57	0.754
Drinking	–5.33	–21.68	11.02	0.521
BMI	1.25	–0.49	2.99	0.156
E2 gene carrying	–22.21	–39.501	–4.925	0.012

CI: confidence interval.

**TABLE 5:** Analysis of factors influencing plaque length following atorvastatin therapy.

	B	B (95%CI) lower limit	B (95%CI) upper limit	*P*
Gender	0.28	–0.25	0.81	0.292
Age	–0.27	–0.05	–0.01	0.028
Hypertension	0.47	–0.05	0.99	0.077
Diabetes mellitus	0.019	–0.47	0.51	0.930
CHD	–0.25	–0.93	0.43	0.466
AF	0.44	–0.65	1.53	0.428
Smoking	0.62	0.13	1.11	0.014
Drinking	–0.42	–1.14	0.31	0.261
BMI	0.01	–0.06	0.09	0.730
E4 gene carrying	0.95	0.29	1.61	0.005

CI, confidence interval.

**TABLE 6:** Multivariate regression analysis of factors influencing the LDL-C reduction rate.

Variables	Unadjusted		Model 1		Model 2	
	B (95%CI)	*P*-value	B (95%CI)	*P*-value	B (95%CI)	*P*-value
LDL-C	–22.21 (–39.50, –4.93)	0.012	–22.94 (–40.24, –5.64)	0.032	–22.51 (–39.47, –5.56)	<0.001
Length of plaque	0.95 (0.29, 1.61)	0.005	0.99 (0.33, 1.65)	0.002	1.06 (0.42, 1.71)	<0.001

For LDL-C, Model 1: adjusted for age and gender. Model 2: adjusted for Model 1 + hypertension, smoking. For the length of the plaque, Model 1: adjusted for age and gender. Model 2: adjusted for Model 1 + diabetes mellitus, coronary heart disease, atrial fibrillation, body mass index.

**TABLE 7:** Changes in vulnerable plaques after 12 months of atorvastatin therapy.

Outcome	APOE genotype group				*P*-value
	Total group *n* = 78	E2 cases *n* = 10	E3 cases *n* = 57	E4 cases *n* = 11	
Effective, no, %. yes	28 (35.9)	7 (70.0)	19 (33.3)	2 (18.2)	0.036
No	50 (64.1)	3 (30.0)	38 (66.7)	9 (81.8)	

## Discussion

This study examined the effects of APOE genetic polymorphisms on atorvastatin therapy in regulating blood lipids and promoting plaque stabilization. Our results showed that compared to ε3 and ε4 allele carriers, ε2 allele carriers had greater lipid-lowering effect on LDL-C, enhanced carotid artery plaque stabilization by atorvastatin, and slower plaque progression.

Polymorphisms of the APOE gene change the structure and function of APOE lipoproteins, resulting in a difference in the affinity of different isomers for LDL receptors and leading to differences in blood lipid levels between carriers of different genotypes. Type E4 preferentially binds to the larger LDL and VLDL receptors, whereas types E2 and E3 preferentially bind to the smaller HDL receptor (18, 19). Additionally, the affinity of the E2 type for LDL receptors is more than 50 times weaker than that of the E3 type, and the binding force of the E4 type to VLDL is strong, which weakens the process of VLDL lipolysis in the peripheral blood (6, 20). Therefore, the APOE genotype may be associated with basal blood lipid levels, and the E4 allele may be associated with higher TC, LDL-C, TG, and VLDL-C and lower HDL-C levels (21). A study found that the LDL-C and TC levels of patients in the E4 carrier group tended to be higher than those of patients in the E2 and E3 groups (22), while a study by Xie et al. showed that the blood TC level of the E4 gene carrier group was significantly higher than that of the E2 and E3 groups (23). Our study also found that the median initial TC level in the E4 group was slightly higher than that in the E2 and E3 groups, suggesting that there may be a correlation between the APOE genotype and basal blood lipid level.

The lipid-lowering effect of statins is closely related to APOE gene polymorphisms. The E4 allele may attenuate the lipid-lowering effect of statins (24), while the E2 allele exerts a relatively better lipid-lowering effect (4). After 3 months of atorvastatin treatment, the effect on LDL-C reduction was highest in the E2 group, followed by the E3 and E4 groups. Although there was no significant difference between the three groups in the TC reduction rate after treatment, the reduction effect of TC was found to be in the following order: E2 group > E3 group > E4 group, indicating that E2 carriers had a higher tendency to lower lipids with statins than other carriers. Some studies have demonstrated that there are sex differences in the lipid-lowering effect of APOE on statins; that is, the E2 allele enhances the lipid-lowering effect of statins, which is more significant in male patients, but not in female patients (25); this may be related to differences in immune activation and hormone levels (26). Consistent with previous findings, our study also found that E2 allele carriers had a greater LDL-C-lowering effect on statins in males, while this difference was not apparent in females.

APOE polymorphisms can affect the occurrence and development of carotid plaques through various mechanisms. First, many previous studies have shown that APOE gene polymorphisms affect blood lipid metabolism, and dyslipidemia is a significant risk factor for the occurrence and development of atherosclerotic plaques. Therefore, APOE may affect the blood lipid metabolic pathway and carotid plaque progression. Second, APOE gene polymorphisms are associated with the development of diabetes (27), but the underlying mechanism is currently unknown. Studies have speculated that E4 carrier status may affect peripheral and central insulin metabolism (28, 29); therefore, differences in blood glucose metabolism can impact arterial plaque development. Third, studies have found that E4 carriers have higher expression of lipoprotein-related phospholipase, which can promote the body’s inflammatory response and plaque instability (30, 31).

Our results provide robust evidence for personalized lipid-lowering and plaque stabilization treatments based on APOE genotypes. This precise treatment can maximize the efficacy of statins. People can achieve lower LDL-C levels, reducing the incidence and recurrence rate of stroke. However, several limitations should also be noted. First, this is a single-center study, which limited the ability to draw major conclusions for all populations. Further studies with larger sample sizes are needed to corroborate our findings. Second, we determined the properties of plaques using ultrasound based on the echo morphology of plaques, which may be less precise than using high-resolution magnetic resonance imaging.

## Conclusion

Polymorphisms of the APOE gene are related to the effects of atorvastatin on lipid lowering and the progression of carotid artery plaques. The population with the ε2 allele experienced a better lipid-lowering effect on LDL-C and slower progression of carotid artery plaques than the population with the ε3 or ε4 allele. Patients with E4 genotype need a higher statin dose or a change to another statin to achieve a better effect. A larger population is required to provide more reliable evidence to explore this relationship.

## Data availability statement

The data analyzed in this study is subject to the following licenses/restrictions: The data are available from the corresponding author on reasonable request. Requests to access these datasets should be directed to biqianqian12@163.com.

## Ethics statement

The studies involving human participants were reviewed and approved by the Ethics Committee of Shanghai Tenth People’s Hospital. The patients/participants provided their written informed consent to participate in this study.

## Author contributions

XZ and JW revised the study. QB drafted the manuscript. QB, YL, and YW analyzed and interpreted the data. QB and WF collected the data. XZ and FW contributed to the critical revision of the manuscript. All authors read and approved the final manuscript.
